# Genetic characteristics of local horse breeds by microsatellite DNA loci

**DOI:** 10.18699/vjgb-25-13

**Published:** 2025-02

**Authors:** N.V. Blohina, L.A. Khrabrova

**Affiliations:** All-Russian Research Institute of Horse Breeding, Divovo, Rybnovsky district, Ryazan region, Russia; All-Russian Research Institute of Horse Breeding, Divovo, Rybnovsky district, Ryazan region, Russia

**Keywords:** Equus caballus, genetic diversity, DNA microsatellites, local breeds, horse, Equus caballus, генетическое разнообразие, микросателлиты ДНК, местные породы, лошадь

## Abstract

Russia has a significant pedigree diversity of horse breeds with unique gene pools that are well adapted to a wide variety of harsh natural and climatic conditions, are characterized by universal performance and high productive qualities, and are of significant interest to the world horse breeding. Genetic studies of population diversity in horse breeding are very relevant, since many domestic horse breeds are under threat of extinction. Biomaterials (hair, blood, semen) from horses of 15 local breeds bred in the Russian Federation and neighboring countries (CIS) were selected for the research. The sample included 2,193 horses, including: Altaiskaya (n = 48), Bashkirskaya (n = 130), Buryatskaya (n = 30), Vyatskaya (n = 220), Zabaikalskaya (n = 34), Kyrgyzskaya (n = 100), Mezenskaya (n = 148), Mugalzharskaya (n = 109), Novoaltaiskaya (n = 514), Pechorskaya (n = 31), Shetland pony (n = 47), Priobskaya (n = 85), Tuvinskaya (n = 600), Khakasskaya (n = 47) and Yakutskaya (n = 50) breeds. The following indicators were used in the genetic and population analysis: the total number of allele variants (Na) in 17 microsatellite loci, the level of polymorphism (Ae), the average number of alleles per locus (Nv), observed (Ho) and expected (He) heterozygosity, coefficients of genetic similarity and genetic distances, as well as the coefficient of intrapopulation inbreeding (Fis). Modern local horse breeds, even relatively small in number, have a high level of biodiversity and a peculiar genetic structure, often with the presence of private alleles, which persists despite periodic crossing with stud breeds of different specializations. It was found that horses of local breeds possess a number of unique alleles, including ASB2T, HMS7S, HMS6J, HMS6H, HMS2T, HMS1O, HTG7L, HTG6L, HTG6H, VHL20S, ASB17Z, ASB17X, ASB17U, LEX3S, LEX3R and CA425E, which were not detected in representatives of stud breeds in the studied European populations. The majority of the studied breeds were characterized by a negative Fis value and the absence of inbreeding. The coefficients of genetic similarity of local breeds varied in a relatively wide range (0.828–0.973) and testified to the uniqueness of the gene pools of most local horse breeds of the Russian Federation, as well as confirmed the common origin of the Kyrgyzskaya horse with the horse populations of Southern Siberia.

## Abstract (ru)

Россия обладает значительным породным разнообразием конских пород с уникальными генофондами, которые хорошо адаптированы к самым разнообразным суровым природно-климатическим условиям, характеризуются универсальной работоспособностью, высокими продуктивными качествами и представляют существенный интерес для мирового коневодства. Генетические исследования популяционного разнообразия в коневодстве являются весьма актуальными, так как многие отечественные породы лошадей находятся под угрозой исчезновения. Для исследований были отобраны биоматериалы (волосы, кровь, сперма) от лошадей 15 местных пород, разводимых в России и сопредельных странах. Выборка включала 2193 лошади алтайской (n = 48), башкирской (n = 130), бурятской (n = 30), вятской (n = 220), забайкальской (n = 34), кыргызской (n = 100), мезенской (n = 148), мугалжарской (n = 109), новоалтайской (n = 514), печорской (n = 31), шетлендских пони (n = 47), приобской (n = 85), тувинской (n = 600), хакасской (n = 47) и якутской (n = 50) пород. При проведении генетико-популяционного анализа использовали следующие показатели: общее количество вариантов аллелей (Na) в 17 микросателлитных локусах, уровень полиморфности (Ae), среднее число аллелей на локус (Nv), наблюдаемая (Ho) и ожидаемая (He) гетерозиготность, коэффициенты генетического сходства и генетических дистанций, коэффициент внутрипопуляционного инбридинга (Fis). Современные местные породы лошадей даже при сравнительно небольшой численности имеют высокий уровень биоразнообразия и своеобразную генетическую структуру, часто с наличием приватных аллелей, которая сохраняется несмотря на периодическое скрещивание с заводскими породами разной специализации. Установлено, что лошади местных пород обладают рядом уникальных аллелей, включая ASB2T, HMS7S, HMS6J, HMS6H, HMS2T, HMS1O, HTG7L, HTG6L, HTG6H, VHL20S, ASB17Z, ASB17X, ASB17U, LEX3S, LEX3R и CA425E, которые не выявлены у представителей заводских пород в изученных европейских популяциях. Для большинства изученных пород было характерно отрицательное значение Fis и отсутствие внутрипородного инбридинга. Коэффициенты генетического сходства местных пород менялись в сравнительно широком диапазоне (0.828–0.973) и свидетельствовали об уникальности генофондов большинства местных конских пород в России, а также подтверждали общность происхождения кыргызской лошади с популяциями лошадей Южной Сибири.

## Introduction

Until the beginning of the last century, horse breeding occupied a special place in the economy and agricultural production not only in Russia, but also in many other countries. The horse was not only a symbol of the power and prestige of the country, but also saved peoples in difficult times of history. However, in the middle of the 20th century, with the development of mechanization in agriculture, transport, army and industry, the approach to the use of horses changed, and the number of horses decreased sharply, but then stabilized and even began to grow in many countries. Now horses play an important role in tourism, sports, racing business, as well as in food production (milk, koumis, meat). The observed global trend of increasing horse meat production (Askarov et al., 2020) is explained by the high dietary properties of horse meat. Horse meat is easily digested and contains practically no allergenic amino acids; therefore, it is considered a valuable dietary product (Stanislawczyk et al., 2020).

World practice shows that if the expenses on breeding work are reduced, the productivity of livestock decreases, and ultimately, animal breeding becomes unprofitable. The assessment of genetic diversity within individual breed populations and entire breeds makes it possible to create and improve both breeding plans and programs for the conservation of the gene pool of these breeds (Marzanov et al., 2010; Kalashnikova et al., 2022). In our country, local horse breeds represent more than 50 % of the total horse population, and their breeding provides employment, livestock production, preservation of cultural traditions and the development of new territories.

**Fig. 1.**
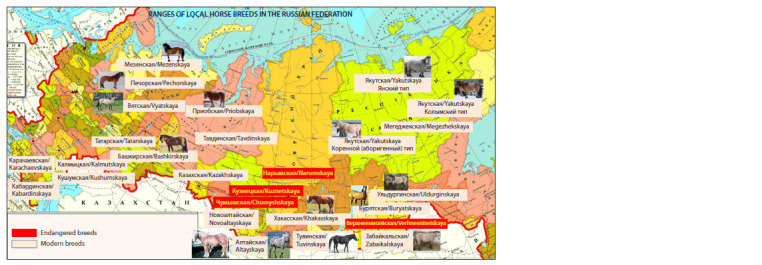
Distribution of horses of local breeds in the territory of the Russian Federation.

Currently, 47 horse breeds are included in the State Register of Breeding Achievements of the Russian Federation, including 20 local and productive breeds created on their basis, which are mainly distributed in regions with harsh climatic conditions (Fig. 1).

The evolution of local horse breeds took place mainly under the influence of natural selection using methods of native breeding, which resulted in high adaptability to environmental conditions. To date, a significant part of the native breeds have been systematically improved by crossing with various stud breeds due to the need to produce a larger horse for agriculture and increase horse meat production. Nowadays, the value of local breeds is increasing, as they are the basis of a productive horse breeding industry (Askarov et al., 2020). As a result of crossing horses of local breeds with stud, mainly draft breeds, it was possible to increase the size of native horses while maintaining their type and economically useful qualities. However, since the end of the last century to the present, there has been no purposeful breeding work with many local breeds, breeding records have been established almost only in the Mezenskaya, Vyatskaya, Bashkirskaya and Kalmykskaya breeds.

Uncontrolled crossing leads to a change in the type and pure original aboriginal forms of horses of most local breeds, which in the future may lead to the disappearance of the breeds themselves. Out of the 40 native horse breeds described by hippologists at the turn of the 19–20th centuries, only 16 have actually survived to the present day. At the same time, there is virtually no information about the current state of the Pechorskaya, Chumyshskaya, Kuznetskaya and Verkhneyeniseiskaya breeds included in the State Register of Breeding Achievements of the Russian Federation (Belousova, 2018).

Table 1 provides information on the number of horses tested and the number of mares of the studied local breeds. It follows from the above data that critically low numbers of livestock are observed in Vyatskaya, Mezenskaya, Pechorskaya and Priobskaya breeds, which are in urgent need of conservation measures. Therefore, comprehensive programs are needed to preserve small-numbered horse breeds, which are a national achievement of Russia, including genetic research for study and evaluation of the valuable domestic gene pool.

**Table 1.**
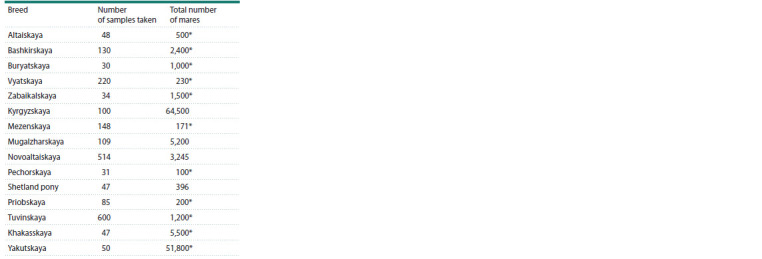
The total number of livestock and the number of samples taken from horses of local breeds * Data: Belousova N.F. (2018).

Today, DNA technologies are widely used in the control of animal origin, the study of phylogenetic relationships and microevolution of breeds, in order to improve the genotypic assessment of animals at individual and population levels, as well as in diverse and genomic studies (Marzanov et al., 2010; Roh et al., 2020; Nwachukwu et al., 2022; Pozharskiy et al., 2023).

In recent years, microsatellites have been the most popular markers in studies of the genetic characteristics of farm animals (Ernst, Zinovieva, 2008; Glazko et al., 2023), they are easy to use and have a high degree of information. Microsatellites are interesting because they are subject to a higher level of mutation than the rest of the genome (Glazko et al., 2023). Studies by many scientists researching the genetic structure of horses of different specializations using DNA markers (Kalashnikov et al., 2011; Blohina et al., 2018; Gavrilicheva, 2019; Khaudov et al., 2019) have shown a high level of allelic variability in the studied populations and confirmed the presence of genetic specificity of the allelofund of most horse breeds, often with a limited breeding area (Vdovina, Yurieva, 2021; Khrabrova et al., 2022). Microsatellites are an effective tool for studying the features of the gene pool, genetic polymorphism, phylogeny, and obtaining data on the formation and evolution of animals (R2D2 Consortium et al., 2021).

The purpose of our study was to investigate the allele pool and genetic diversity of STR loci in the genomes of horses of 15 native breeds bred in the territory of the Russian Federation and CIS countries, as well as to study their phylogenetic relationships.

## Materials and methods

The materials for these studies were selected from representatives of local breeds living on the territory of the Russian Federation. The study included 2,193 horses, including: Altaiskaya (n = 48), Bashkirskaya (n = 130), Buryatskaya (n = 30), Vyatskaya (n = 220), Zabaikalskaya (n = 34), Kyrgyzskaya (n = 100), Mezenskaya (n = 148), Mugalzharskaya (n = 109), Novoaltaiskaya (n = 514), Pechorskaya (n = 31), Shetland pony (n = 47), Priobskaya (n = 85), Tuvinskaya (n = 600), Khakasskaya (n = 47) and Yakutskaya (n = 50) breeds.

The studies were conducted in the certified laboratory of genetics of the Federal State Budgetary Scientific Institution All-Russian Research Institute for Horse Breeding for 17 STR DNA loci: HMS2, HMS3, HMS1, AHT4, VHL20, AHT5, HTG7, HTG6, HTG4, HTG10, HMS7, HMS6, ASB23, ASB2, ASB17, LEX3 and CA425, using standardized techniques recommended by ISAG.

DNA isolation from biomaterials (hair, blood, sperm, etc.) was carried out using COrDIS SPRINT reagents (Russia). Amplification of the obtained DNA was performed using a 17-plex set of primers for genotyping horses of domestic production COrDIS Horse (Russia). The separation and detection of amplification products were carried out by capillary electrophoresis on an automatic 4-capillary genetic analyzer NANAFOR 05 (Russia). After recording the electrophoresis data using the GeneMapper™ V.4.0 program, the sizes of the amplified DNA fragments were calculated. The interpretation of the results was carried out using a control DNA profile with a known genotype and data from international comparative tests (Horse Comparison Tests) conducted by ISAG in 2008–2020. An international alphabetic code was used to designate alleles. The analysis of the genetic and population parameters of the breeds was carried out and graphically visualized in the program R Studio 1.3.1093 (Francis, 2017), R package “diveRsity”, using the package “POPHELPER”. The expected (Ho) and observed (He) heterozygosity values were calculated using the PLINK 1.9 software packages (Chang et al., 2015); MS Excel 2010, Statistics 12 (https://stat soft-statistica.ru/) and GenAlEx (ver.6.5.1) (https://biologyassets. anu.edu.au/GenAlEx/Download.html) were also used in the calculations. The phylogenetic tree was constructed using the Neighbor-Net algorithm using the SplitsTree4 4.14.5 program (https://www.advanceduninstaller.com/SplitsTree4- 4_14_5-72c0418345e4a971ba5b353bfae970d6-application. htm).

When characterizing the breeds, the following indicators were calculated: the total number of alleles in 17 STR loci (Na), the average number of alleles per locus (Nv), the level of polymorphism (Ae), observed (Ho) and expected (He) heterozygosity, the coefficient of intrapopulation inbreeding (Fis), genetic kinship and genetic distances

## Results

Genotyping of 2,193 horses of 15 local breeds at 17 STR loci revealed 521 alleles with large fluctuations in loci from 3 for HTG6 (Pechorskaya) to 21 for ASB17 (Tuvinskaya).

A comparative analysis of the generalizing indicators shows that the richest spectrum of alleles was recorded in horses of the Tuvinskaya (170), Novoaltaiskaya (158) and Mugalzharskaya (154) breeds, while horses of the Buryatskaya breed had the minimum number of alleles, which was 117 (Table 2).

**Table 2.**
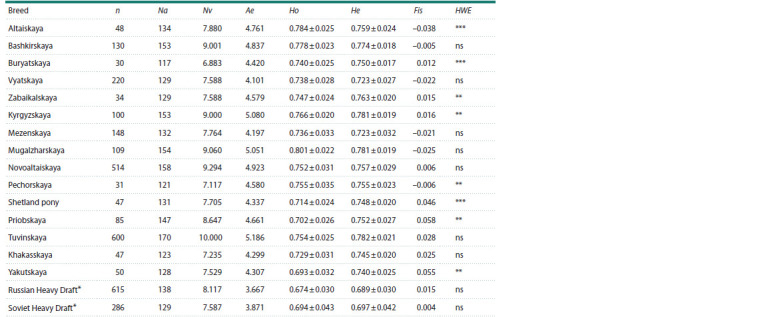
Characteristics of horses of native breeds (n = 2,193) according to 17 STR DNA markers Notе. n – number of horses; Na – total number of alleles in 17 microsatellite loci; Ae – level of polymorphism; Ho – observed heterozygosity; He – expected heterozygosity; Fis – coefficient of intrapopulation inbreeding; Nv – average number of alleles per locus; HWE – deviation from the Hardy–Weinberg equilibrium (ns – not significant, ** – significant at p <0.001, *** – significant at p <0.0001), * – horse breeds participating as improvers of local horses.

The Tuvinskaya breed was the best in terms of polymorphism (Ae = 5.186). Horses of the Kyrgyzskaya (Ae = 5.080) and Mugalzharskaya (Ae = 5.051) breeds had similar indicators of this value. The lowest level of polymorphism was recorded in horses of the Khakasskaya (Ae = 4.299) and Yakutskaya (Ae = 4.307) breeds. The highest indicators of actual heterozygosity were determined in horses of the Mugalzharskaya (Ho = 0.801), Altayskaya (Ho = 0.784), Bashkirskaya (Ho = 0.778) and Kyrgyzskaya (Ho = 0.766) breeds, the lowest degree was found in Yakutskaya (Ho = 0.693) horses.

The predominance of heterozygous genotypes and the absence of intrapopulation inbreeding were observed in horses of the Altaiskaya, Bashkirskaya, Vyatskaya, Mezenskaya and Mugalzharskaya breeds. A slight shift in the genetic balance towards an excess of homozygotes according to the Fis coefficient was noted in horses of the Zabaikalskaya, Kyrgyzskaya, Novoaltaiskaya, Priobskaya, Tuvinskaya, Khakasskaya and Yakutskaya breeds. In most subpopulations and breeds of horses, we observed deviations from the Hardy–Weinberg equilibrium, significant at p < 0.001 or p < 0.0001 for all studied loci. The Hardy–Weinberg equilibrium was observed in horses of Bashkirskaya, Vyatskaya, Mezenskaya, Mugalzharskaya, Novoaltaiskaya, Tuvinskaya, and Khakasskaya breeds (p > 0.05).

The results of the analysis of the genetic structure of horses of native breeds demonstrate that each of the analyzed groups differs in the spectrum, frequency of occurrence and set of alleles. It should be noted that a comparative analysis of 17 STR loci in horses of local breeds revealed 16 new alleles that were missing from the standardized ISAG nomenclature (Van de Goor et al., 2010), namely alleles: HMS7S, HMS6J, HMS6H, HMS2T, HMS1O, ASB2T, HTG7L, HTG6L, HTG6H, ASB17Z, ASB17X, ASB17U, VHL20S, LEX3S, LEX3R and CA425E.

In horses of the Mezenskaya breed bred in the Arkhangelsk region, five unique alleles were found at once: HMS6J (0.003), ASB17Y (0.019), ASB17X (0.010), LEX3S (0.039) and LEX3R (0.016). The alleles HMS7L (0.685), HMS3M (0.432), AHT4O (0.417), HTG7O (0.437), HTG7K (0.425), HTG6O (0.799), HTG4M (0.419) and LEX3M (0.535) had the highest concentration in this northern forest breed.

In the genetic structure of the Bashkirskaya breed, there is a high frequency of occurrence of certain alleles (HTG10O – 0.447, HTG6O – 0.508, HTG4M – 0.589 and HMS7L – 0.487) and the presence of a rare allele ASB17U (0.041), found in horses of Tuvinskaya breed.

Two alleles turned out to be unique alleles for horses of the Vyatskaya breed HTG6L (0.004) and AHT5P (0.009), and the typical ones were HMS7L (0.470), HMS2H (0.457), HMS1M (0.468), AHT5J (0.427), HTG7 (0.576), HTG6O (0.712) and HTG4M (0.689).

The local horse breeds of Siberia differed markedly from European populations in all genetic parameters. High values of all basic population parameters were determined in horses of the Kyrgyzskaya and Tuvinskaya breeds that previously had contiguous ranges. High allele frequencies were also identified in horses of these breeds: HTG6O (0.548; 0.530), HTG4M (0.632; 0.597), HMS7L (0.420; 0.400). The Tuvinskaya breed rarely had alleles HMS6H (0.001), HMS3L (0.004), VHL20K (0.002), ASB23N (0.001), ASB17Z (0.002), LEX3J (0.054) and LEX3I (0.024). Two unique alleles were identified in the Kyrgyzskaya horse breed, HTG4J (0.005) and HTG7Q (0.009), absent from other studied breeds within the studied groups

The HTG6R allele was detected only in horses of four breeds: Kyrgyzskaya (0.005), Mugalzharskaya (0.007), Priobskaya (0.023) and Tuvinskaya (0.006). At the VHL20 locus, the S allele was found in horses of the Mugalzharskaya (0.046), Novoaltaiskaya (0.005) and Tuvinskaya (0.002) breeds.

**Fig. 2.**
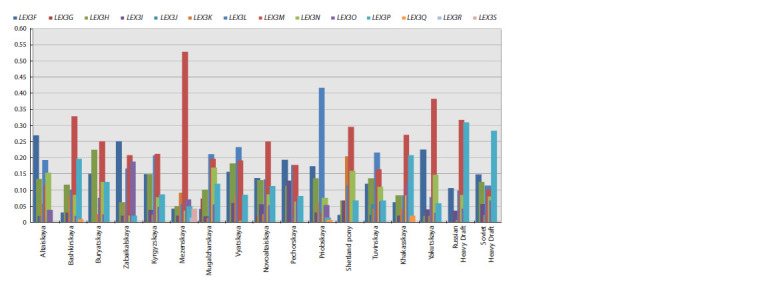
Histogram of allele frequencies at the LEX3 locus in horses of local breeds.

In the LEX3 locus localized on the X chromosome, 12 alleles were identified in local horses, three of which (F, L, M) were found in all the studied breeds (Fig. 2).

Representatives of the Tuvinskaya and Altaiskaya breeds are characterized by the presence of a rare allele LEX3J (0.019; 0.056), which is absent in other groups studied in this work. Only in horses of local breeds bred in Southern and Western Siberia, such as Kyrgyzskaya, Novoaltaiskaya, Mugalzharskaya and Tuvinskaya, the LEX3G allele was found, which occurred with low frequency

Rare alleles have been identified in the genotypes of horses of the Mezenskaya breed, LEX3S (0.045) and LEX3R (0.017), missing from other populations (Fig. 2).

Horses of local breeds were characterized by the presence of separate alleles at the CA425 locus (I, J, L, M and N). The maximum frequency of occurrence of the CA425M allele was detected in the Tuvinskaya and Khakasskaya populations, and the CA425N allele was determined in horses of the Buryatskaya (0.511), Altaiskaya (0.463), Mugalzharskaya (0.408) and Novoaltaiskaya (0.435) breeds. The new allele CA425E was found only in horses of the Mugalzharskaya breed bred in Kazakhstan (0.009). The unique CA425P allele was identified in Shetland ponies, Bashkirskaya and Khakasskaya horses, and was absent in other breeds we studied.

The indicators of the level of polymorphism and the degree of heterozygosity in local breeds were high at low Fis values, which indicates a genetic balance in the studied populations. Based on the results obtained, it can be noted that the highest indicators of genetic diversity were found in horses of the Tuvinskaya breed, in comparison with other breeds analyzed in the framework of the presented study. And the lowest level of genetic diversity is observed in horses of the Vyatskaya breed.

The coefficients of genetic kinship between local horse breeds varied in the range of 0.828–0.973 (Table 3). The highest coefficients of genetic relationship were determined in horses of the Kyrgyzskaya breed with the Tuvinskaya (0.973), Bashkirskaya (0.939), Altaiskaya (0.938), Zabaikalskaya (0.934) and Khakasskaya (0.926) breeds. The lowest level of genetic relationship was found with Shetland ponies. Genetic differences between the studied horse breeds by microsatellite markers confirm the values of genetic distances, which varied in the range of 0.027–0.331. Horses of the Tuvinskaya and Kyrgyzskaya breeds have the closest genetic distances (0.027).

**Table 3.**
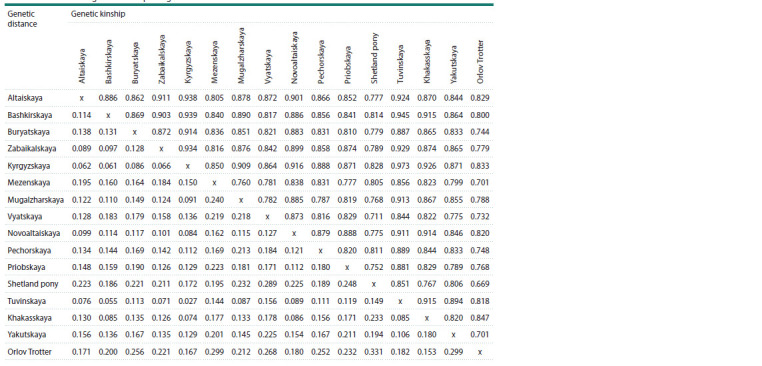
Coefficients of genetic kinship and genetic distances in horses of local breeds

**Fig. 3.**
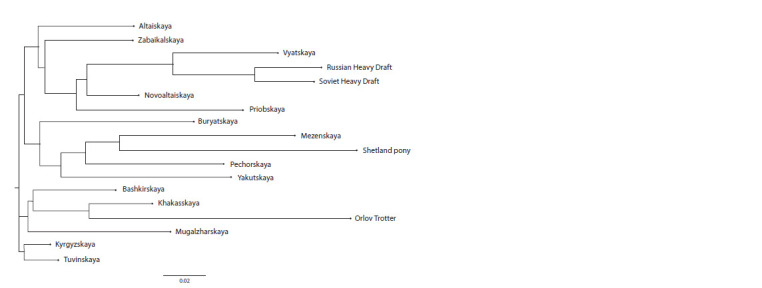
Dendrogram of genetic distances between native horse breeds, constructed using the Neighbor-Net method

On the phylogenetic tree, the studied horse breeds form three independent clusters (Fig. 3). The first cluster includes horses of the Kyrgyzskaya and Tuvinskaya breeds, characterized by a common origin, which is adequately consistent with history. Ethnic groups actively roamed on horseback throughout Eastern, Western and Central Siberia, hence the genetic relationship of the populations.

The second cluster is formed by horses of the Bashkirskaya, Khakasskaya, Mugalzharskaya and Orlov Trotters breeds. At first glance, the inclusion of the Orlov Trotters in the group of local steppe breeds looks somewhat unexpected, but most likely it is due to the periodic use of this trotter breed to improve the economically useful qualities of horses of local populations. In this subcluster, a branch of the geographically isolated Mugalzharskaya breed stands out somewhat, possessing a number of unique alleles

The most extensive third cluster includes most of the studied aboriginal horse breeds of the forest and forest-steppe zone, as well as breeds of domestic draft horses previously used as improvers of local livestock. The dendrogram of genetic distances clearly reflects the noticeable influence of stallions of the Russian draft breed on the formation of the Vyatskaya breed over the past decades of its development.

Thus, based on the results obtained, it can be concluded that the formation of genetic profiles of aboriginal breeds, their levels of polymorphism, differences in the structure of loci, genetic and population characteristics, as well as commonalities, are influenced by two groups of significant factors of different vectors: the natural geographical isolation of animals and origin from common ancestors. Our molecular genetic analysis of 15 native breeds showed that all animal groups were characterized by high genetic diversity.

## Discussion

Russia has unique genetic resources of horse breeding, the study of biological characteristics of which is the basis for programs for their conservation and improvement.

In the course of the conducted studies in horses of native breeds, high values of the polymorphism level of STR markers were determined in almost all breeds (Ae = 4.101–5.186) analyzed in the framework of the presented study. The data obtained indicate a complex and diverse system of crossing and breeding in the studied groups, as well as the presence of genetic diversity associated with the adaptive qualities of horses and their ability to adapt to extreme environmental conditions in an evolutionary context.

In addition to the standardized nomenclature (Van de Goor et al., 2010), 16 new alleles were found in horses of local breeds, which could have remained in the centers of domestication of ancient horses in the territory, as well as appeared as a result of genomic mutations or the introduction of genes with horses of nomads from different regions of Asia. Scientists from China (Ling et al., 2011) confirm this fact with their own studies of local Chinese horses, in which a wide range of alleles of microsatellite markers of oriental origin has been identifie

The analysis revealed significant differences in the main genetic parameters (Ae, Nv, Ho, He, Fis). In addition to the high degree of genetic variability, a characteristic feature of local breeds was the presence of a number of alleles unique for domestic breeds (ASB2T, HMS7S, HMS6J, HMS6H, HMS2T, HMS1O, HTG7L, HTG6L, HTG6H, VHL20S, ASB17Z, ASB17X, ASB17U, LEX3S, LEX3R and CA425E), which were not found in horses of stud breeds and in the studied European populations (Seo et al., 2016; Baena et al., 2020).

Horses of native breeds have unique alleles: Tuvinskaya – HTG6L, VHL20S, HMS6H, ASB17X, ASB17U, ASB17Z; Bashkirskaya – ASB17U; Altaiskaya – HMS2T; Buryatskaya – HTG6L, HTG6H; Vyatskaya – AHT5P, HTG6L; Mezenskaya – ASB17Y, ASB17X, HMS6J, LEX3R, LEX3S. Modern aboriginal horse breeds, even with a common origin from Mongolian roots (Yun et al., 2022), have their own characteristic genetic structure with the presence of private alleles, despite periodic crossing with stud breeds of riding, trotting and draft directions.

Our results confirm the published data of foreign scientists (Lippold et al., 2011; Librado et al., 2021) regarding the area of horse domestication having occupied a significant part of modern Russia, which, due to its geographical location, was a historical crossroads of the routes of many nomadic peoples of Eurasia, which contributed to the intensive process of forming horses of new breeds.

## Conclusion

Thus, the conducted studies have shown that domestic horse breeds have an original genetic structure, an inherent allele pool and are characterized by a high level of genetic diversity. Private alleles have been identified in horses of native breeds, which must be taken into account when controlling the origin and assessing population diversity, as well as when conducting genetic monitoring and planning programs for the conservation and breeding of horses of local breeds

The results of the constructed phylogenetic tree show that local horse breeds bred in the territories of neighboring regions have the highest degree of genetic similarity. Cluster analysis combined horse breeds into three groups according to the genetic structure of DNA microsatellite loci, which confirmed their suitability as markers of phylogenetic relationship of populations. The obtained coefficients of genetic similarity adequately reflect the relationships of local horse breeds in accordance with the history of their formation.

The study of the features of the genetic structure and phylogenetic relationships of domestic aboriginal horse breeds by 17 STR markers is of undoubted interest both from a theoretical and a practical point of view. Genetic breeding methods make it possible not only to assess the degree of genetic diversity of breeds, but also to control the level of inbreeding, and based on this to form a strategy for breeding programs.

## Conflict of interest

The authors declare no conflict of interest.
